# Musculoskeletal manifestations in mucopolysaccharidosis type I (Hurler syndrome) following hematopoietic stem cell transplantation

**DOI:** 10.1186/s13023-016-0470-7

**Published:** 2016-07-08

**Authors:** Mona Schmidt, Sandra Breyer, Ulrike Löbel, Sinef Yarar, Ralf Stücker, Kurt Ullrich, Ingo Müller, Nicole Muschol

**Affiliations:** Department of Pediatrics, University Medical Center Hamburg-Eppendorf, Martinistraße 52, 20246 Hamburg, Germany; Department of Pediatric Orthopedics, Altonaer Children’s Hospital, Bleickenallee 38, 22763 Hamburg, Germany; Department of Diagnostic and Interventional Neuroradiology, University Medical Center Hamburg-Eppendorf, Martinistraße 52, 20246 Hamburg, Germany; Department of Trauma, Hand and Reconstructive Surgery, University Medical Center Hamburg-Eppendorf, Martinistraße 52, 20246 Hamburg, Germany; Clinic of Pediatric Hematology and Oncology, Division for Stem Cell Transplantation, University Medical Center Hamburg-Eppendorf, Martinistraße 52, 20246 Hamburg, Germany

**Keywords:** Mucopolysaccharidosis type I, Hurler syndrome, Musculoskeletal manifestations, Craniocervical stenosis, Odontoid hypoplasia, Thoracolumbar kyphosis, Hip dysplasia, Dysostosis multiplex

## Abstract

**Background:**

Hematopoietic stem cell transplantation (HSCT) is the treatment of choice for young Hurler patients. Despite halting of neurocognitive decline and improvement of life expectancy, the beneficial effect on the skeletal system is limited. As orthopedic complications are one of the most disabling factors following HSCT, this points to the need for new treatment strategies. The study summarizes musculoskeletal manifestations in 19 transplanted Hurler patients.

**Methods:**

Data were obtained retrospectively. Patients’ charts for physical examinations of the joint range of motion (JROM) of shoulders, elbows, hips and knees were reviewed. Radiographic evaluations of thorax, spine, pelvis and hands were performed. MRI scans of the craniocervical junction were analyzed to determine odontoid hypoplasia and the prevalence of craniocervical stenosis.

**Results:**

Nineteen Hurler patients (10 females, 9 males) with an average age of 8.1 years (range 2.5–23.8) at the latest follow-up, who underwent allogenic HSCT between 1991 and 2012, were assessed after an average follow-up period of 6.4 years (range 0.7–22.5). Seventeen patients achieved long-term engraftment, two developed graft failures. The majority of patients showed a steady state or improvements in the mobility of knees (31 %/63 %), hips (47 %/40 %) and elbows (56 %/38 %). However, shoulder abduction was impaired in ¾ of patients and showed the highest rate of progression (31 %). In patients with graft failure, progressive restrictions in JROM were noted. Assessments of the craniocervical junction by MRI showed stable or improved diameters in 67 % of patients. Correction or stabilization of odontoid hypoplasia was found in 64 %. However thoracolumbar kyphosis, scoliosis, hip dysplasia and genua valga were progressive despite HSCT. At the last follow up, 47 % of patients were partially wheelchair dependent, 10 % wheelchair bound and 25 % regularly experienced pain in the spine, hips and lower extremities due to orthopedic problems.

**Conclusion:**

Joint mobility, odontoid hypoplasia and craniocervical stenosis might stabilize or even improve in Hurler patients following HSCT. However, despite the beneficial effects on some musculoskeletal manifestations, skeletal complications are frequently observed and the overall burden of orthopedic disease is significant. Frequent multi-disciplinary follow-up in a specialized center are essential. Novel therapeutic approaches (e.g. anti-inflammatory drugs) are needed to improve musculoskeletal outcomes.

**Electronic supplementary material:**

The online version of this article (doi:10.1186/s13023-016-0470-7) contains supplementary material, which is available to authorized users.

## Background

Mucopolysaccharidosis (MPS) type I is an autosomal recessive inherited metabolic disorder caused by a deficiency of lysosomal α-iduronidase (IDUA, EC 3.2.1.76). Consequently, the glycosaminoglycans (GAGs) heparan- and dermatan-sulfate accumulate within the lysosomes causing cellular dysfunction [1, 2]. The incidence of MPS I in Germany has been estimated as 0.69 cases per 100,000 births [3]. Clinical severity and progression of the disease vary significantly in affected patients. Based on the clinical appearance three different subtypes of MPS I are classified: the classical severe phenotype referred to as Hurler syndrome (MPS I-H), the less severe Hurler-Scheie syndrome (MPS I-H/S) and the attenuated form - Scheie syndrome (MPS I-S) [1, 2, 4]. Clinical symptoms of Hurler syndrome include the characteristically coarse facial features, corneal clouding, hearing impairment, cardiac involvement, obstructive and restrictive pulmonary disease and hepatosplenomegaly. With increasing age patients also show progressive neurological decline. Musculoskeletal involvement is common in all subtypes of MPS I. Skeletal abnormalities include: a short stature, degenerative joint disease and a skeletal dysplasia referred to as dysostosis multiplex [5, 6].

The pathophysiology of the bone disease is still not well understood. Disordered growth plate chondrocyte organization and trabecular architecture were observed in several animal models of different MPS subtypes [7]. As shown in studies in MPS I mice, accumulation of GAGs could lead to inactivation of the major osteoclastic protease cathepsin K, which in turn may result in reduction in cartilage degradation and contribute to the bone pathology observed in MPS [8]. The progressive arthropathy is assumed to be mediated by GAG accumulation, resulting in increased cytokine and chemokine recruitment (e.g. TNF-alpha, IL-1-beta) and leads to increased apoptosis of chondrocytes, synovial hyperplasia and inflammatory joint destruction - despite the lack of clinical inflammation signs [9, 10].

Craniocervical stenosis (CCS) with consecutive spinal cord compression and neurological symptoms such as paraplegia are known features of severe MPS I-H and can lead to significant disability [11]. Narrowing of the cervical spinal canal at the craniocervical junction (CCJ) is presumed to originate from bony stenosis, peri-odontoid soft tissue masses, hypertrophy of the anterior and posterior longitudinal ligaments and dural thickening [11–13]. Furthermore atlanto-axial instability due to odontoid hypoplasia can lead to spinal cord compression and myelopathy [11, 14, 15]. In addition, MPS I patients have a high risk of developing a carpal tunnel syndrome (CTS) due to bone dysplasia as well as thickening of synovia and ligaments [16–19]. Early recognition and treatment of CTS are important to protect severe limitations in hand acquirements. Diagnosis of CTS can be difficult because of the absence of characteristic clinical symptoms such as nocturnal pain, numbness or tingling [19, 20].

Therapeutic options in MPS I are hematopoietic stem cell transplantation (HSCT) and, since 2003, disease-specific enzyme replacement therapy (ERT) with recombinant α-iduronidase [21]. HSCT is the treatment of choice in Hurler patients ≤2.0 years of age with a developmental quotient (DQ) of >70 [5] and has altered the natural history of MPS I-H dramatically, leading to clinical improvements and the halting of neurocognitive decline. Skeletal manifestations, on the other hand, have remained progressive [11, 22–24]. Due to the available therapies, life expectancy and survival have improved significantly in patients with MPS I, accentuating the need for new concepts to treat the orthopedic manifestations. However, there is a lack of knowledge concerning the impact of the available treatments on the skeletal system [25, 26].

The purpose of this study was a longitudinal evaluation of muculoskeletal manifestations in 19 Hurler patients following hematopoietic stem cells transplantation.

## Methods

All patients attended the MPS outpatient clinic at the University Medical Center Hamburg-Eppendorf and were monitored and examined between 1991 and 2014. Nineteen patients (9 male, 10 female) with an average age of 8.1 years (range 2.5–23.8 years) at the latest follow-up met the inclusion criteria: diagnosis of MPS I confirmed by enzyme activity determination and/or genetic testing, Hurler phenotype, hematopoietic stem cell transplantation (HSCT) and a minimum of two orthopedic evaluations.

All available data before HSCT, as well as changes of musculoskeletal manifestations following HSCT, were collected retrospectively by reviewing patient charts, radiographs of thorax, spine, pelvis and hands as well as magnetic resonance imaging (MRI) scans of the craniocervical junction (CCJ). Furthermore, any surgical interventions for musculoskeletal disease were analyzed. HSCT was carried out at an average age of 1.6 years (1.1–2.2 years), follow-up after HSCT ranged between 0.7 and 22.5 years. All data were anonymized before analysis.

### Joint mobility

Physical examinations of joint range of motion (JROM) of shoulders, elbows, hips and knees recorded in the patient charts were reviewed. The following movements were analyzed based on the clinical relevance: shoulder abduction, elbow extension, hip extension and knee extension. Joint contractures were categorized as mild, moderate and severe. Restriction of shoulder abduction was classified as mild (deficit ≤40°), moderate (deficit of 50–80°) or severe (deficit ≥90°). Flexion joint contractures of elbows, hips and knees were classified as mild (5–15°), moderate (≥15°) or severe (≥25°). The course of joint disease was analyzed a minimum of six months after HSCT.

#### Odontoid hypoplasia and craniocervical stenosis

Odontoid hypoplasia and the prevalence of craniocervical stenosis (CCS) were analyzed by MRI. To evaluate odontoid hypoplasia an odontoid process/odontoid body ratio was calculated as described previously [27]. A ratio below 2 was termed hypoplasia. The measurements were done on 3–4 mm sagittal T2-weighted images using a 1.5 Tesla MRI scanner. In addition, axial and coronal T2- or T1-weighted sequences were used for identification of the odontoid, if available. To quantify the CCS the smallest anterior-to-posterior diameter of the CCJ (vertebrae C1-C3) was measured. An anterior-to-posterior diameter of less than 14 mm was defined as a cervical stenosis [28]. Patient charts were reviewed for surgical decompression of the CCS or stabilization in case of atlanto-axial instability.

#### Thoracolumbar kyphosis and scoliosis

Kyphotic angle and degree of scoliosis were determined by radiograph of the thoracolumbar spine. The measurements were performed by standardized technique from end vertebra to end vertebra and were specified by Cobb angle assessments in the sagittal plane [29].

#### Hip dysplasia

Radiographic evaluations of the hips were performed on anterior-posterior pelvic radiographs with hips in neutral rotation and a supine position. Hip dysplasia was quantified by the acetabular index (AI). According to the method of Toennies and Brunken [30] acetabular dysplasia was specified by an AI ≥2 standard deviations (SD). Furthermore, the Reimers’ head migration index (MI) was used to classify the hip joint stability into normal (<25 %), subluxation (33–99 %) and dislocation (100 %) [31]. The records of patients who underwent surgical interventions were reviewed to identify the age at intervention, as well as the type and number of surgeries.

#### Further signs of dysostosis multiplex

Dysostosis multiplex is characterized by abnormalities of skeletal features including thorax, spine, pelvis, limbs and hands [1, 2, 5]. Any available radiographs were reviewed to detect the prevalence of these distinct abnormalities.

## Results

### Patients

Nine male and ten female Hurler patients with an average age of 8.1 years (range 2.5–23.8 years) at the latest follow-up fulfilled the inclusion criteria. Genetic testing was available in 17 patients. In two patients diagnosis was confirmed by enzymatic testing and the clinical phenotype only. The study population is summarized in Table 1.

**Table 1:** Study population

No.	Gender (m/f)	Year of birth	Age at HSCT (y)	Follow-up after HSCT (y)	Age at latest follow-up (y)	Chimerism at latest follow-up (%)	Genotype			
							1. Allele (cDNA)	1. Allele (Protein)	2. Allele (cDNA)	2. Allele (Protein)
1	f	2010	2.0	0.7	2.7	99.9	c.1205G > A	p.W402X	c.1205G > A	p.W402X
2	f	2010	1.5	2.0	3.5	94	c.606c > A	p.Y202X	c.979G > C	p.A327P
3	f	2009	1.3	1.9	3.2	100	n.a	n.a.	n.a.	n.a.
4	f	2009	2.2	2.3	4.5	16.7	c.208C > T	p.Q70X	c.1205G > A	p.W402X
5	m	2008	1.8	4.2	6.0	99.9	c.979G > C	p.A327P	c.1099G > C	p.A367P
6	m	2008	1.5	3.2	4.7	98	c.208C > T	p.Q70X	c.175delA (fs132X)	-
7	f	2008	2.0	2.0	4.0	99.9	c.208C > T	p.Q70X	c.208C > T	p.Q70X
8	m	2008	1.5	4.6	6.0	59.3	c.208C > T	p.Q70X	c.208C > T	p.Q70X
9	m	2008	2.2	4.2	6.4	100	c.208C > T	p.Q70X	c.1045_1047delGAC	p.D349del
10	f	2007	1.2	5.7	6.9	99.9	c.208C > T	p.Q70X	c.1413C > G	p.Y471X
11	f	2007	1.6	5.0	6.6	99.9	c.1205G > A	p.W402X	c.1205G > A	p.W402X
12	f	2006	1.9	2.7	4.6	99.9	c.917G > A	p.W306X	c.1727 + 5G > A	n.i.
13	m	2005	1.5	7.8	9.3	99.9	c.208C > T	p.Q70X	c.1205G > A	p.W402X
14	m	2002	1.1	10.9	12.0	5.4	c.1205G > A	p.W402X	c.1205G > A	p.W402X
15^a^	m	2002	1.1	10.3	11.4	0	c.1205G > A	p.W402X	c.1205G > A	p.W402X
16	f	2002	1.8	10.6	12.4	99.9	c.979G > C	p.A327P	n.i.	n.i.
17	m	1999	1.8	13.3	15.2	50.5	c.208C > T	p.Q70X	c.979G > C	p.A327P
18	f	1996	2.1	7.9	10.0	30	n.d.	n.d.	n.d.	n.d.
19	m	1990	1.2	22.5	23.8	n.d.	c.1205G > A	p.W402X	c.1205G > A	p.W402X

*m* male, *f* female, *y* years, *n.a* not available, *n.i*. not identified, *n.d* not done

^a^Patient was treated with ERT after graft failure

Hematopoietic stem cell transplantation (HSCT) was carried out at an average age of 1.6 years (1.1–2.2 years); follow-up after HSCT ranged between 0.7 and 22.5 years (average 6.4 years). Patients 3, 6 and 9 were transplanted in other centers; all others received HSCT at the Stem Cell Transplantation Unit at the University Medical Center in Hamburg. Five patients received enzyme replacement therapy (ERT) prior to HSCT (patients 1, 2, 3, 6 and 9). Fourteen patients received HSCT from an HLA-identical unrelated donor, while two patients were transplanted from HLA-identical family donors (patients 18 and 19). Two patients (patients 5 and 11) were transplanted from HLA-mismatched unrelated donors and patient 1 received HSCT from a haplo-identical family donor. The most commonly used conditioning regime (*n* = 9, 47 %) was Fludarabine, Busulfan, Melphalan and anti-thymocyte globulin (ATG) (patients 3, 5, 6, 7, 8, 10, 11, 12 and 13); followed by Cyclophosphamide, Busulfan and ATG (*n* = 6, 32 %; patients 14, 15, 16, 17, 18 and 19). Patients 1 and 9 were re-transplanted after graft failure. In these cases the age at the time of the second transplantation was used for further analysis. Twelve patients presented with full donor chimerism (≥94 % donor cells), while four patients had mixed chimerism with 16.7 to 59.3 % of donor alleles between 2.3 and 4.6 years post HSCT. In patient 19, chimerism was not part of the standard diagnostic routine at the time of transplantation, but enzyme activity analyses were always within the normal range. Patient 15 developed a graft failure within the first year after transplantation. As the family decided not to carry out a second transplantation, he was treated with ERT. Patient 14 presented with a slow decline in donor chimerism over 10.9 years of follow-up and was not re-transplanted or treated with ERT. Ten patients developed acute Graft-versus-Host-Disease (aGvHD) following transplantation. Patient 5 developed chronic hepatopathy of unknown origin. GvHD of the liver was excluded by liver biopsy.

### Clinical review

Orthopedic clinical evaluations were available for all patients and were carried out between an average age of 2.1 years (range 0.7–4.5) at the first evaluation and 8.2 years (range 2.7–23.8) at the last evaluation. Repeated examinations were available in 18 patients with an average follow-up period of 6.2 years (range 1.4–21.5).

**Fig. 1:**
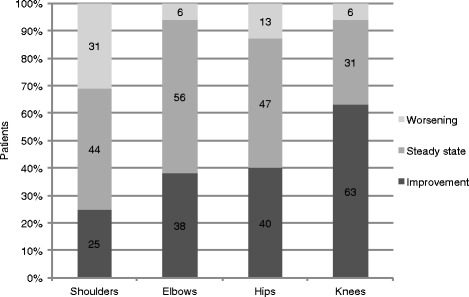
Course of joint mobility in successfully transplanted MPS I patients (*n* = 17). Change in grade of severity (assessed in categories as mild, moderate severe) of shoulder abduction as well as elbow, hips and knee extension at the last available follow up is presented. The highest percentage of improvement was seen in knees (63 % of patients), hips (40 % of patients) and elbows (38 % of patients)

All patients had limited shoulder abduction at some point during follow-up, generally combined with flexion contractures in other joints. The majority of successfully transplanted patients showed a steady state or even improvements in joint range of motion (JROM) following HSCT (Fig. 1). At the most recent follow-up there was no extension deficit found in the knees, 69 % (11/16), hips, 53 % (8/15) and elbows, 44 % (7/16), of the patients. However, in 31 % (5/16) of patients shoulder mobility worsened, leading to severe restrictions. The two patients with graft failure (patients 14 and 15) experienced progressive contractures in almost all joints. The improvement/worsening was symmetrical in all cases. Detailed results of the physical examinations of JROM over time are shown in Table 2.

**Table 2:** Restriction of joint range of motion

No.	Age at evaluation (y)	Follow-up after HSCT (y)	Shoulders	Elbows	Hips	Knees
1	0.7	0	mild	mild	moderate	severe
	2.7	0.7	severe	none	mild	moderate
2	1.1	0	severe	none	mild	none
	2.5	1.0	severe	mild	mild	mild
	3.5	2.0	severe	mild	n.d.	n.d.
3	3.2	1.9	severe	mild	none	mild
4	1.9	0	moderate	mild	none	mild
	2.1	0.2	moderate	none	none	none
	4.1	1.8	mild	none	none	none
	4.5	2.3	severe	none	none	none
5	1.5	0	severe	none	mild	none
	3.9	2.1	severe	mild	mild	mild
	5.1	3.3	n.d.	mild	n.d.	mild
	6.0	4.2	severe	mild	none	none
6	1.3	0	none	none	none	none
	3.5	2.0	mild	none	none	none
	4.7	3.2	mild	none	n.d.	none
7	1.9	0	severe	mild	moderate	none
	3.0	1.0	mild	moderate	moderate	mild
	4.0	2.0	mild	none	mild	none
8	1.2	0.3	none	none	moderate	none
	4.0	2.5	mild	mild	moderate	mild
	5.1	3.6	mild	mild	n.d.	mild
	6.0	4.6	moderate	none	none	none
9	3.0	0.8	none	none	none	none
	3.9	1.7	mild	none	mild	none
	6.4	4.2	none	mild	mild	none
10	2.2	1.0	moderate	mild	none	mild
	3.4	2.1	moderate	mild	mild	mild
	6.0	4.7	moderate	mild	none	none
11	2.6	1.0	none	none	none	none
	5.1	3.5	mild	mild	none	mild
	6.6	5.0	severe	mild	none	mild
12	1.8	0	severe	moderate	none	none
	4.0	2.1	moderate	moderate	severe	mild
	4.6	2.7	moderate	mild	moderate	mild
13	1.0	0	severe	none	none	none
	2.5	1.0	n.d.	none	none	mild
	9.3	7.8	moderate	none	none	none
14	1.7	0.7	moderate	mild	none	mild
	9.6	8.5	severe	mild	moderate	mild
	10.4	9.4	severe	severe	severe	mild
	12.0	10.9	severe	severe	severe	severe
15	1.7	0.6	severe	moderate	mild	mild
	7.0	5.9	severe	severe	severe	mild
	10.7	9.6	severe	severe	severe	mild
	11.4	10.3	moderate	moderate	severe	mild
16	1.5	0	mild	mild	none	mild
	6.9	5.1	mild	mild	none	none
	11.8	9.9	mild	mild	none	none
	12.4	10.6	mild	mild	none	none
17	3.9	2.1	mild	mild	none	mild
	8.1	6.3	mild	mild	n.d.	mild
	12.9	11.0	mild	mild	mild	none
	15.2	13.3	mild	mild	moderate	none
18	2.7	0.6	n.d.	mild	n.d.	mild
	7.2	5.1	mild	mild	mild	mild
	8.8	6.7	severe	mild	n.d.	mild
19	2.3	1.0	n.d.	mild	n.d.	mild
	7.2	6.0	n.d.	mild	severe	mild
	11.4	10.2	mild	none	none^a^	none
	19.8	18.6	mild	none	none^a^	none
	23.8	22.5	mild	none	mild^a^	none

Shoulder abduction was classified as mild (deficit ≤40°), moderate (deficit of 50–80°) or severe (deficit ≥90°)

Flexion joint contractures of elbows, hips and knees were classified as mild (5–15°), moderate (≥15°) or severe (≥25°)

*y* year, * n.d.*not done

^a^Status after hip release surgery

Thoracolumbar kyphosis was clinically diagnosed in 89 % (17/19) of patients at a mean age of 1.3 years and was the first orthopedic symptom of the disease in most cases. In 58 % (11/19) a combination of kyphosis and scoliosis was found. Onset of kyphosis was observed 2.4 years earlier than the onset of scoliosis (mean age 3.7 years). Pectus excavatum was found in nine patients (mean age 3.5 years) and did not significantly progress during the follow-up period.

Limitations in hand function, such as fist clenching and flexion deformities especially in distal interphalangeal joints (DIJ) and ulnar deviation, were common features. The prevalence of carpal tunnel syndrome (CTS) was 71 % (12/17). In two patients there was no data on nerve conduction velocity available. Eleven patients underwent bilateral open carpal tunnel release between 1.6 and 9.4 years of age. Only patient 16 required a revision three years after the initial surgery.

**Fig. 2:**
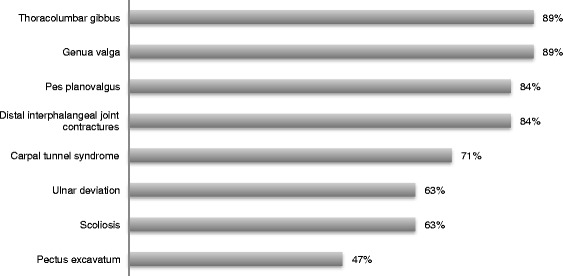
Overall prevalence of orthopedic manifestations at any time point over the course of disease

Genua valga were found in 89 % (17/19) of patients and were first identified at a mean age of 3.2 years. Progression was noted in all cases. Five patients (patients 4, 7, 12, 16 and 17) underwent correction with eight-plates between 3.4 and 11.1 years of age. Patient 17 required a second surgery after recurrence of the deformity. Patient 19 was treated with three osteotomies at 8, 11 and 13 years of age. Pes planovalgus appeared to be the most common foot deformity (16/19). The overall prevalence of orthopedic manifestations are summarized in Fig. 2.

### Imaging review

#### Cervical spine

MRI scans of the craniocervical junction (CCJ) were available in 17 patients and were carried out between an average age of 2.5 years (range 0.8–15.3) at the first evaluation and 8.7 years (range 3.4–23.8) at the most recent follow-up.

In the initial MRI the smallest CCJ diameters ranged from 9 to 11 mm in 10 patients, while 6 patients presented with a diameter below 9 mm (patients 5, 6, 12, 14, 15 and 17) which was associated with reduced cerebrospinal fluid (CSF) spaces around the cervical spinal cord. Nevertheless, there were no signs of myelopathy. By contrast, patient 1 presented with severe spinal cord compression (CCJ diameter 3 mm) and floppy paraplegia at 9.6 months of age and underwent surgical decompression twice.

**Fig. 3:**
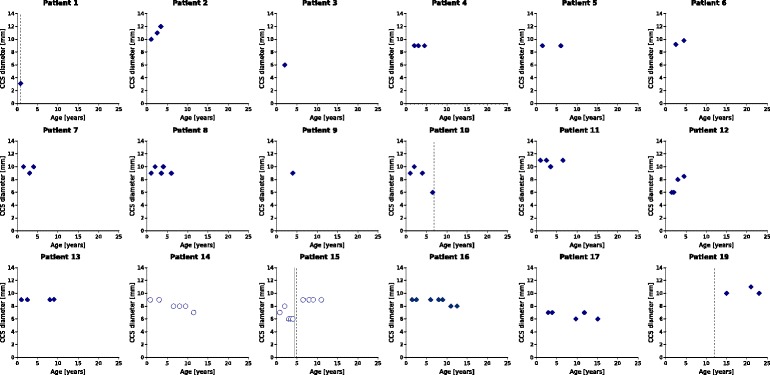
Longitudinal MRI assessments of CCJ diameters. The shortest anterior-to-posterior diameters of the CCJ (vertebrae C1-C3) were assessed on sagittal T2-weighted MRI scans and are shown for each patient separately. The two patients with graft failure (patient 14 and 15) are presented as circles. *Dashed straight lines* indicate the time of surgical intervention. The *straight line* represents the start of ERT

Serial MRI scans were available for 15 patients. In 20 % (3/15) of patients the CCJ diameter improved slightly (between 1 and 3 mm) over an average follow-up period of 2.3 years (range 1.8–2.8). In 46 % (7/15) stable diameters between 7–11 mm were measured at an average follow-up period of 5.5 years (range 2.1–10.9). In four patients (patients 10, 14, 15 and 17) craniocervical stenosis (CCS) was progressive. Images of patient 14 and 17 did not reveal signs of myelopathy during follow-up. Patient 10 and 15 developed symptomatic CCS (CCJ diameter 6 mm) at 6.9 and 4.9 years respectively and were treated with surgical decompression. Patient 19 also underwent cervical decompression at 12.0 years of age for symptomatic CCS; a pre-operative MRI was not available for review. Assessments of CCJ diameters over time are shown in Fig. 3.

Odontoid hypoplasia, as defined above, was observed in 65 % (11/17) of the patients in the initial MRI study. Four patients (patients 4, 8, 12 and 16) showed an improvement of the odontoid morphology, while in three patients (patients 5, 13 and 17) the odontoid remained unchanged. Patient 14, who experienced graft failure, showed progressive odontoid hypoplasia. Patient 15, who also developed graft failure, had an odontoid/body ratio of 1.3 and developed atlanto-axial instability with indication for surgical C1-2 fusion at the age of 4.9 years. After surgery and the initiation of ERT, odontoid hypoplasia improved. In two patients (patients 1 and 9) only one MRI scan was available. In contrast to all other patients, patient 19 presented with a proportionally smaller body of the dens compared to the odontoid process, resulting in a ratio >2. This patient was surgically treated with C1-2 fusion due to atlanto-axial instability at 12.0 years of age. Unfortunately, pre-operative MRI scans were not available for review. A normal odontoid process was observed in five cases (patients 2, 6, 7, 10 and 11). All patients presented with increased soft tissue mass surrounding the odontoid.

#### Thoracolumbar spine

Lateral radiographs of the thoracolumbar spine in standing position were available for 17 patients. In thirteen patients with an average age of 2.6 years (range 0.8–7.8) at the first evaluation, thoracolumbar kyphosis ranged from 10° to 90°. While in four patients (patients 1, 2, 7, and 8), thoracolumbar kyphosis was absent at an average age of 1.6 years (range 1.1–2.0). Nine of ten patients with an available follow-up showed progression of kyphosis of 8° to 57° over an average follow-up period of 4.8 years (1.1–12.8). In patient 14 the kyphotic angle remained unchanged. In patient 15, who developed graft failure, the first available radiograph at the age of 7.8 years revealed a kyphosis with a Cobb angle of 90°. This patient subsequently underwent dorsal spondylodesis. Patient 19 was treated with dorsal spondylodesis due to symptomatic kyphosis at 14.3 years of age. Pre-operative x-rays were not available for review.

Anterior-posterior spinal x-rays in standing position were available for 16 patients. Mild scoliosis was found in eleven patients with a deformity ranging between 7° and 35°. Only patient 14, who had graft failure, presented with moderate scoliosis (Cobb angle 40°). Overall, eight patients were treated with braces due to kyphosis and/or scoliosis. Patient 16 developed segmental instability between L2 and L3 despite bracing at 10.8 years of age. Surgical intervention is indicated but has not as yet been performed. Longitudinal measurements of kyphotic and scoliotic angles are shown in Table 3.

**Table 3:** Measurements of kyphosis and scoliosis

No.	Age at evaluation (y)	Follow-up after HSCT (y)	Kyphotic angle (°)	Scoliotic angle (°)	Therapy
1	1.7	0	0	23	none
2	1.1	0	0	0	none
	3.4	1.9	n.d.	0	
3	1.2	0	30	n.d.	Brace
	2.2	0.9	38	n.d.	
4	2.1	0	12	35	none
5	1.5	0	14	0	none
	3.8	2.0	33	0	
6	0.8	8	37	n.d.	Brace
7	2.0	0	0	0	none
8	1.4	0	0	0	
	2.5	1.0	45	n.d.	Brace
	3.6	2.1	52	0	
	5.9	4.5	57	7	
9	4.3	2.0	10	28	Brace
	5.1	2.9	14	24	
	5.7	3.5	20	19	
10	2.2	1.0	n.d.	0	none
	3.3	2.1	n.d.	0	
	4.4	3.2	n.d.	0	
11	1.3	0	30	0	none
	3.7	2.1	n.d.	0	
12	1.4	0	28	8	none
	4.6	2.7	62	16	
13^a^	1.5	0	45	9	none
	2.5	1.0	45	9	
	8.1	6.6	73	10	
14	0.9	2.4	30	n.d.	Brace
	9.6	8.6	32	40	
15	7.8	6.8	90	n.d.	Dorsal spondylodesis at 7.8 y
16	4.2	2.4	17	9	
	5.2	3.4	27	n.d.	
	6.4	4.5	32	15	
	6.9	5.1	40	n.d.	Brace
	10.8	8.9	n.d.	22	
17	1.0	0	30	n.d.	
	4.5	2.7	40	n.d.	
	6.5	4.7	58	n.d.	Brace
	12.5	10.7	56	19	
	13.8	12.0	55	15	
18	6.2	4.1	60	n.d.	
	8.8	6.7	80	16	
	10.0	7.9	72	n.d.	Brace
19^b^	13.5	12.3	n.a.^b^	7	Dorsal spondylodesis at 14.3 y

*y* year, *n.d*.not done, *n.a* not available

^a^No continuous follow-up was performed as patient lived abroad. Bracing treatment planned

^b^Pre-operative x-rays not available for review

#### Hips

Radiographs of the hips were available in 16 patients with an average age of 4.3 years (range 0.7–11.2) at the first evaluation. Acetabular dysplasia was demonstrated in all cases except one (patient 1). Due to hip contractures the acetabular index (AI) could not be assessed in two patients (patients 8 and 14). In 13 of 14 patients who could be analyzed (93 %) acetabular dysplasia (AI ≥ 2 SD) was found in both hips. In patient 1 hip dysplasia was absent at 0.7 years of age. Follow-up x-rays were available for five patients and showed progressive hip dysplasia over a follow-up period of 3.2 years (range 0.3–7.6). Twelve of the 16 patients (75 %) developed bilateral femoral head subluxation (average MI left hip 62.5 % ± 15.4; right hip 59.3 % ± 15.9). Patient 14, who developed graft failure, presented with bilateral femoral head dislocation (MI both hips 100 %). Three patients (patients 1, 4 and 12) presented with normal head migration indices. Hip containment surgery was carried out in four cases (patients 4, 10, 17 and 19) at a median age of 6.3 years (range 3.9–11.4). Three patients underwent Pemberton acetabuloplasty, while in one patient Dega acetabuloplasty was performed.

#### Further signs of dysostosis multiplex

The presence of dysostosis multiplex was assessed by examination of x-rays of the spine, thorax, pelvis and hands (Additional file 1: Figure S1). Flattened and beaked vertebrae, as well as paddle shaped ribs, were found in all patients (19/19). Broad iliac wings were observed in all patients with available x-rays of the hips (16/16). All patients with available hand x-rays (11/11) presented with typical manifestations of dysostosis multiplex of the hands, such as hypoplastic and irregular carpal bones (11/11), proximal pointing of metacarpals (10/11), shortened phalanges and metacarpals (9/11), as well as a V-shaped hypoplastic distal ulna and radius (9/11). Reduced calcification of the bones of the hand was noted in all cases (11/11).

## Discussion

The present study gives a systematic overview of the prevalence, progression and treatment of orthopedic manifestations in 19 patients with Hurler syndrome after HSCT. Due to the improved prognosis and survival rate there is an increasing need for more detailed analysis of the skeletal system involvement in such patients, in order to develop more effective treatment strategies.

In Hurler syndrome, restriction in the joint range of motion (JROM) of upper and lower limbs can be observed early in life and may lead to severe disability [1, 2, 5]. Deposition of GAGs and inflammatory processes are thought to be responsible for joint disease [7, 9, 32, 33]. In the present study joint stiffness in elbows, hips and knees did not progress or even improved in some of the transplanted Hurler patients. The highest percentage of improvement was seen in the knees (63 % of patients) followed by hips (40 % of patients) and elbows (38 % of patients). In prior studies the positive effects of HSCT on joint mobility were documented in elbows, knees and also shoulders of MPS I patients [22–25]. However, in our patient population shoulder abduction was impaired in all patients and showed the highest rate of progression (31 %) over time. Seven patients in our cohort presented with severe restrictions of shoulder mobility from the first available evaluation and only two of those (25 %; patients 7 and 12) showed any improvement over time. This is in contrast to previously published data by Weisstein et al. 2004 [25] and Souillet et al. [23] who observed improvement of shoulder mobility in 71 % (5/7) and 85 % of patients, respectively. Irreversible shoulder joint pathology prior to transplantation as a result of inflammation of the capsule [34, 35], could explain our results, however, the discrepancy with previous results cannot be explained.

Our study identified carpal tunnel syndrome (CTS) in 12 patients (71 %). CTS is a common symptom of MPS I, caused by bone dysplasia, a thickening of the flexor retinaculum and GAG deposition within the carpal tunnel [16, 20]. Eleven patients, who were treated with open carpal tunnel release and tenosynovectomy at the University Medical Center Hamburg-Eppendorf achieved functional improvement of hand dexterity and remained stable during a follow-up period of 1.6 to 6.1 years. Patient 16 needed revision three years later but was stable during a follow up of four years. CTS relapse did not appear to be a common complication in this patient group. Although there are no standards for the diagnosis and treatment of CTS in MPS patients, a surgical release is recommended in MPS patients. However, there is no consensus about the optimal time for this surgery and a lack of long-term outcome data [19, 20]. Khanna et al. [18] suggested that early HSCT may decrease the risk of CTS. However, in our study population no correlation between the incidence of CTS and the age at transplantation could be identified.

Hip dysplasia was found in all but one patient, who was 8 months old at the time of evaluation. Progression was found in all cases with available follow-up data. Hip containment surgery was offered to four patients after a careful clinical evaluation. Diagnostic assessments of soft tissue and cartilaginous coverage of the hips by MRI and arthrograms as well as general health, clinical symptoms, rate of disease progression and individual risk for surgery and anesthesia were taken into account. The prevalence of hip dysplasia is in accordance with findings in the literature [6, 26, 36]. However, there is no agreement on the indication for surgical treatment in these patients [36] and efforts to reach an international consensus have been made, but have so far not been reached due to a lack of evidence [36, 37].

The majority of patients (89 %) presented with progressive genua valga. Epiphyseodesis by eight-plate implantation was a reliable and safe method to correct the deformity. Overcorrection might be indicated, as recurrences are common [38, 39].

In the present study almost 90 % of patients presented with early onset and progressive thoracolumbar kyphosis (mean age 1.3 years). However, only two patients (patients 15 and 19) needed surgical intervention at 7.8 and 14.3 years of age, respectively. Muscle hypotonia and underdeveloped paraspinal muscles as well as ossification abnormalities of the vertebral bodies are assumed to be responsible for the development of kyphosis in MPS diseases [6, 26, 40]. Stoop et al. [41] described early onset of thoracolumbar kyphosis and significant progression despite HSCT. Abelin and colleagues [42] found severe progressive thoracolumbar gibbus in 16 out of 72 MPS I patients, which led to surgical treatment in 14 patients. Therefore, kyphosis should be carefully monitored in transplanted MPS I patients in order to avoid spinal cord compression.

Eleven patients presented with odontoid hypoplasia in the first available MRI, and 36 % (4/11) improved during an average follow-up of 5.2 years (2.3–10.6) after transplantation. By contrast, the appearance of the dens did not change on follow-up in both patients with graft failure (patients 14 and 15). However, patient 15 showed significant improvement of dens hypoplasia three years after atlanto-axial stabilization and initiation of ERT, suggesting that the improvement might be related to ERT. The prevalence of odontoid hypoplasia is in alignment with the findings of previous studies [14, 15, 41].

Cervical spinal cord compression is one of the most feared complications in Hurler syndrome. Studies on CCS have been carried out in MPS I, IVA and VI patients [26, 28, 43]. No documentation of CCS progression in Hurler patients have been published to date. The majority of patients in this study (81 %) presented with asymptomatic stenosis of the CCJ, which stabilized or even improved during follow-up after HSCT. An anterior-to-posterior spinal canal parameter of less than 12 mm is assumed to be associated with clinical symptoms of cord compression [28]. In our study myelopathy, as assessed by MRI and/or somatosensory evoked potential (SEP), was not observed in patients with spinal canal diameters between 9–12 mm. Furthermore, even in some patients with diameters between 6–9 mm and impaired CSF signal, no signs of cord compression were found. On the other hand, patients 10 and 15, with a spinal canal diameter of 6 mm, developed neurological symptoms and signs of myelopathy and required decompression surgery. CCJ measurements are highly dependent on the MRI sequences used (slice thickness, tissue contrast, orientation) and therefore might not be comparable between different centers. The spinal canal diameters should only serve as one of several tools (along with clinical neurological examination, SEP and sleep laboratory evaluation) for assessing the need for a surgical intervention.

A limitation of this study is the retrospective design and the use of different transplantation regimes. However, there was no correlation between the type of conditioning regime or the type of donor and the clinical manifestation of musculoskeletal symptoms. Due to the small sample size, and the different variables assessed at different ages and follow-up time points, statistical interpretability of the data is limited. Overall we were not able to identify any obvious correlation between the different parameters that might influence the clinical outcome. There was no significant difference in the incidence or severity of orthopedic manifestations in patients after early HSCT (≤18 months of age) or late HSCT (>18 months). Physical examinations of the JROM are subjective assessments and therefore classification of the severity of joint restrictions may vary among physicians. Assessment of AI and MI in hip imaging is dependent on correct positioning and may be inaccurate due to hip contractures. No MRI-based norm values for odontoid lengths in children exist at present. CT studies revealed ossification of the odontoid might be incomplete up to the age of 8–14 years [44]. Therefore a delay of ossification in MRI studies might be misinterpreted as odontoid hypoplasia. Although CT is the technique of choice to estimate the ossification rate of the CCJ it is ethically not justifiable in pediatric patients due to high radiation exposure.

## Conclusion

In conclusion, successful HSCT can benefit some musculoskeletal manifestations in Hurler syndrome, as stabilization or even improvement in the joint mobility of elbows, hips and knees was found in the majority of transplanted Hurler patients. However, shoulder abduction was restricted in ¾ of patients and progressive in 31 %. Partial correction of odontoid hypoplasia, as well as prevention of progressive craniocervical stenosis, following HSCT has an important impact on the overall clinical outcome. Nevertheless, thoracolumbar kyphosis, scoliosis, hip dysplasia and genua valga are progressive despite HSCT and may require conservative orthopedic or surgical intervention. Furthermore, the overall burden of orthopedic disease is significant. Walking distance was impaired in almost all of the patients and the majority was not able to perform a 6-minute-walk-test (6MWT) due to muscle weakness and/or pain. At the last follow up, 47 % of patients were partially wheelchair dependent, 10 % wheelchair bound and 25 % of patients regularly experienced pain in the spine, hips and lower extremities due to orthopedic problems. Surgical treatment for orthopedic complications was needed in almost 60 % of patients. A multi-disciplinary team including metabolic specialists, pediatric orthopedists, orthopedic surgeons, neurosurgeons and physiotherapists is essential to prevent musculoskeletal complications. New therapeutic strategies based around the use of anti-inflammatory drugs (e.g. anti-TNF-alpha antibodies, pentosan polysulfate) for the treatment of joint disease may be promising approaches [10, 33, 45].

## Abbreviations

6MWT, 6-minute-walk-test; aGVHD, acute Graft-versus-Host-Disease; AI, acetabular index; ATG, anti-thymocyte globulin; CCJ, craniocervical junction; CCS, craniocervical stenosis; CSF, cerebral spinal fluid; CTS, carpal tunnel syndrome; DIJ, distal interphalangeal joints; DQ, developmental quotient; ERT, enzyme replacement therapy; GAGs, glycosaminoglycans; HSCT, hematopoietic stem cell transplantation; IDUA, α-iduronidase; JROM, joint range of motion; MI, migration index; MPS, mucopolysaccharidosis; MRI, magnetic resonance imaging; SD, standard deviation; SEP, somatosensory evoked potential

## Additional file

Additional file 1: Figure S1. (a-d): Radiographs of dysostosis multiplex. (a) Anterior-posterior spine x-ray of patient 14 at 9.6 years of age: scoliosis (40°) and paddle shaped ribs. (b) Lateral spine x-ray of patient 12 at 4.6 years of age: thoracolumbar gibbus (62°), flattened and beaked vertebrae. (c) X-ray of the left hand of patient 14 at 7.8 years of age: hypoplastic and irregular carpal bones, proximal pointing of metacarpals, shortened phalanges and metacarpals and V-shaped hypoplastic distal ulna and radius, decalcification of the bones of the hand. (d) Anterior-posterior x-ray of patient 4 at 3.4 years of age: broad iliac wings, hip dysplasia. (PNG 804 kb)
