# Supplementary figures and images for: Musculoskeletal manifestations in mucopolysaccharidosis type I (Hurler syndrome) following hematopoietic stem cell transplantation
Source: Orphanet J Rare Dis. 2016 Jul 8;11:93. doi: 10.1186/s13023-016-0470-7 (PMC4938899; doi:10.1186/s13023-016-0470-7)

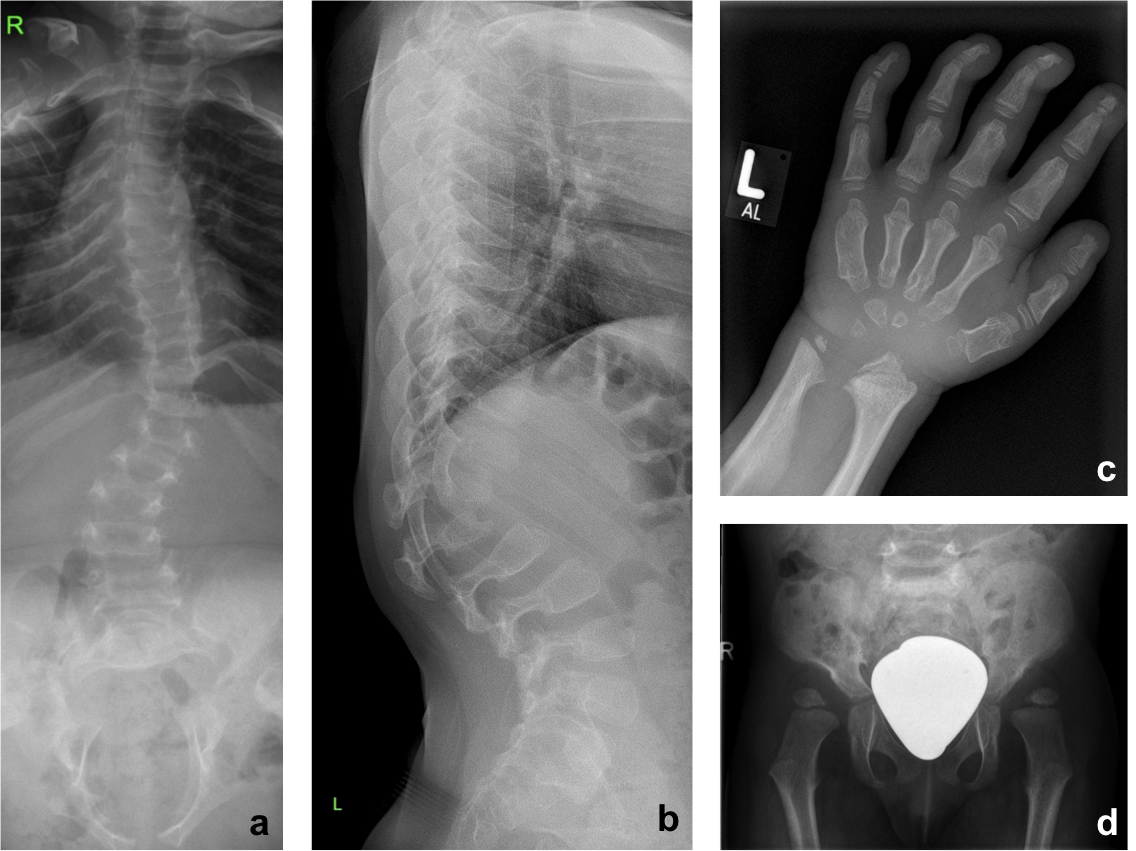

Supplement: Additional file 1: Figure S1. — (a-d): Radiographs of dysostosis multiplex. (a) Anterior-posterior spine x-ray of patient 14 at 9.6 years of age: scoliosis (40°) and paddle shaped ribs. (b) Lateral spine x-ray of patient 12 at 4.6 years of age: thoracolumbar gibbus (62°), flattened and beaked vertebrae. (c) X-ray of the left hand of patient 14 at 7.8 years of age: hypoplastic and irregular carpal bones, proximal pointing of metacarpals, shortened phalanges and metacarpals and V-shaped hypoplastic distal ulna and radius, decalcification of the bones of the hand. (d) Anterior-posterior x-ray of patient 4 at 3.4 years of age: broad iliac wings, hip dysplasia. (PNG 804 kb) [file 13023_2016_470_MOESM1_ESM.png]
